# Genome-wide Analysis of Four *Enterobacter cloacae* complex type strains: Insights into Virulence and Niche Adaptation

**DOI:** 10.1038/s41598-020-65001-4

**Published:** 2020-05-18

**Authors:** Areeqa Mustafa, Muhammad Ibrahim, Muhammad Asif Rasheed, Sumaira Kanwal, Annam Hussain, Asma Sami, Raza Ahmed, Zhu Bo

**Affiliations:** 1 School of Agriculture and Biology, Shanghai Jiao Tong University/Key Laboratory of Urban Agriculture by Ministry of Agriculture of China, Shanghai, 200240 China; 2 Genomics and Computational Biology Laboratory, Department of Biosciences, COMSATS University Islamabad, Sahiwal Campus, Sahiwal, Pakistan; 3 Department of Biotechnology, COMSATS University Islamabad, Abbottabad Campus, Pakistan

**Keywords:** Bacterial genomics, Bacteriology

## Abstract

*Enterobacter cloacae* complex (Ecc) species are widely distributed opportunistic pathogens mainly associated with humans and plants. In this study, the genomes of clinical isolates including *E. hormaechei, E. kobei*, and *E. ludwigii* and non-clinical isolate including *E. nimipressuralis* were analysed in combination with the genome of *E. asburiae* by using the reference strain *E. cloacae* subsp. *cloacae* ATCC 13047; the Ecc strains were tested on artificial sputum media (ASM), which mimics the host, to evaluate T6SS genes as a case study. All five Ecc strains were sequenced in our lab. Comparative genome analysis of the Ecc strains revealed that genes associated with the survival of Ecc strains, including genes of metal-requiring proteins, defence-associated genes and genes associated with general physiology, were highly conserved in the genomes. However, the genes involved in virulence and drug resistance, specifically those involved in bacterial secretion, host determination and colonization of different strains, were present in different genomic regions. For example, T6SS accessory and core components, T4SS, and multidrug resistance genes/efflux system genes seemed vital for the survival of Ecc strains in various environmental niches, such as humans and plants. Moreover, the ASM host-mimicking growth medium revealed significantly high expression of T6SS genes, including PrpC, which is a regulatory gene of the T6SS, in all tested Ecc strains compared to the control medium. The variations in T6SS gene expression in ASM vs. control showed that the ASM system represents a simple, reproducible and economical alternative to animal models for studies such as those aimed at understanding the divergence of Ecc populations. In summary, genome sequencing of clinical and environmental Ecc genomes will assist in understanding the epidemiology of Ecc strains, including the isolation, virulence characteristics, prevention and treatment of infectious disease caused by these broad-host-range niche-associated species.

## Introduction

The *Enterobacter* genus was reported in 1960 by Hormaeche and Edwards^1^. The List of Prokaryotic Names with Standing in Nomenclature (LPSN) reports that 12 species and 2 subspecies are included in this genus. Among them, the species of the *Enterobacter cloacae complex* (Ecc) are opportunistic and can cause lower respiratory tract infections and bacteraemia^2,3^. These bacteria are also notorious for causing nosocomial infections in intensive care units. It has also been reported that The human gastrointestinal tract is the most common endogenous reservoir for these bacteria^4,5^. More recently, it has been noted that Ecc bacteria pose a threat to plants by causing internal decay of onion and yellowing disease of papaya and lucerne seeds in China^6^.

Molecular and biochemical analyses have shown that the Ecc contains 6 species: *E. cloacae subsp. cloacae, E. asburiae*, *E. kobei, E. hormaechei*, *E. ludwigii* and *E. nimipressuralis*. Little is known about the genome correlation among the Ecc strains and the frequency of diseases caused by them compared to other genera in the family of Enterobacteriaceae including Yersinia, Salmonella and Escherichia^5^. The members of the Ecc differ in their pathogenicity to humans, and some members have also been reported to cause epidemic outbreaks^6^.

Prior studies have reported that Ecc strains possess various potential virulence factors, such as secretion systems and multiple drug resistance efflux systems. Among them, the most recently discovered and widely distributed in gram-negative bacteria is the type VI secretion system (T6SS). The T6SS is important for bacterial competition as well as virulence in many gram-negative bacteria, and its dynamics and regulation vary significantly between species. The presence of the T6SS is likely to provide enhanced advantages to Ecc strains in competition with other microbes, consequently allowing the strains to survive in diverse niches^7^. Genetic clusters of T6SSs comprise a group of tightly clustered genes; among these, 13 conserved genes are the core components encoded by both pathogenic and non-pathogenic bacteria.

In order to look into the genetic makeup of the T6SS in Ecc, genome-wide prediction of Ecc genomes followed by gene expression profiling could be vital to understand the physiology of these bacteria isolated from plant and clinical niches^7^. However, genome-wide analysis, including the characterization of specific virulence genes and antimicrobial resistance genes, has only been fully conducted for a few Ecc members.

The aim of the current study is to perform genome-wide analysis of four strains, namely, *E. nimipressuralis* CIP 104980 (non-clinical), *E. hormaechei* ATCC 49162 (sputum of a male patient), *E. ludwigii* EN-119 (clinical) and *E. kobei* JCM 8580 (clinical), sequenced in our lab and to test, optimize and develop an ASM medium to characterize Ecc species using T6SS genes as a case study.

## Results and Discussion

### Features of *Enterobacter cloacae* complex genomes

Genome sequencing of *E. asburiae* revealed a single circular chromosomal DNA of 4.81 Mbp in addition to 4 plasmids. Its genome encodes 4,827 genes, 86 tRNAs and 25 rRNAs, with a GC content of 55.47%^8^. The genome sequence of *E. hormaechei* consists of 3 scaffolds of 4.89 Mbp along with 1 plasmid. Its genome encodes 4,797 genes, 95 tRNAs and 28 rRNAs, with a GC content of 55.1% (Table 1). The draft genome sequence of *E. kobei* possesses a single chromosomal DNA and a plasmid collectively of 4.75 Mbp. This genome encodes 4,626 genes, 89 tRNAs, and 24 rRNAs, with a GC content of 55.43%. The draft genome sequence of *E. ludwigii* consists of a single chromosomal DNA and a plasmid collectively of 4.95 Mbp, encoding 4,795 genes, 88 tRNAs and 29 rRNAs, with a GC content of 55.43% (Table 1). The draft genome sequence of *E. nimipressuralis* was 4.98 Mbp, comprising 18 scaffolds of DNA and encoding 4,875 genes, 93 tRNAs and 30 rRNAs, with a GC content of 55.1%.

**Table 1:** Genomic features of *Enterobacter* strains analysed in this study.

	*E. asburiae*	*E. hormaechei*	*E. kobei*	*E. ludwigii*	*E. nimipressuralis*	*E. cloacae subsp. cloacae* ATCC 13047,
Origin	Human	Human	Human	Human	Plant	Human
Chromosome No.	1	3 scaffolds	1	1	18 scaffolds	1
Plasmid No.	4	1	1	1	Unknown	2
Genome Size (Mb)	4.81	4.89	4.75	4.95	4.98	5.60
Coverage	85×	68×	65×	69×	78×	NA
Coding Genes.	4,827	4,797	4,626	4,795	4,875	5,581
G + C Content (%)	55.47	55.10	55.43	54.60	55.98	54.8
tRNA No.	86	95	89	88	93	84
rRNA No.	25	28	24	29	30	25

The five strains were sequenced by using PacBio technology, and the longest possible sequence of excellent quality and accuracy was obtained. It was further reasoned that PacBio, with a high sequencing coverage, could produce a long sequence read with an enhanced quality. To explore the Ecc genomes and their genetic components, comparative genomics was performed as a fundamental strategy. The identification of genes and other functional elements, such as regulatory regions, and their influence on the survival of the organism depends on revealing the natural selection signature of the genome^9^. We anticipate that the depth of these genomes will be a key factor in exploring the epidemiology of Ecc strains.

The draft genome sequence of *E. cloacae* subsp. *cloacae* ATCC 13047, which was used as the reference genome, had a slightly lower GC content (54.8%), while the number of coding sequences (5,581) of this genome was higher than those of the other sequenced genomes. Ecc species are abundant in nature and are notorious for causing various diseases. However, many biochemical and molecular studies have shown heterogeneity in Ecc species^9^. This has revealed variation in the genetic physiology of this pathogen, including the GC content, number of genes and RNAs. Genomes with low GC contents indicated recurrent horizontal gene transfer in the Ecc strains. The variation in the genomes may be due to the origin of species from various niches and the role of genes that are specific to different niches.

### Comparative genome analysis

**Figure 1:**
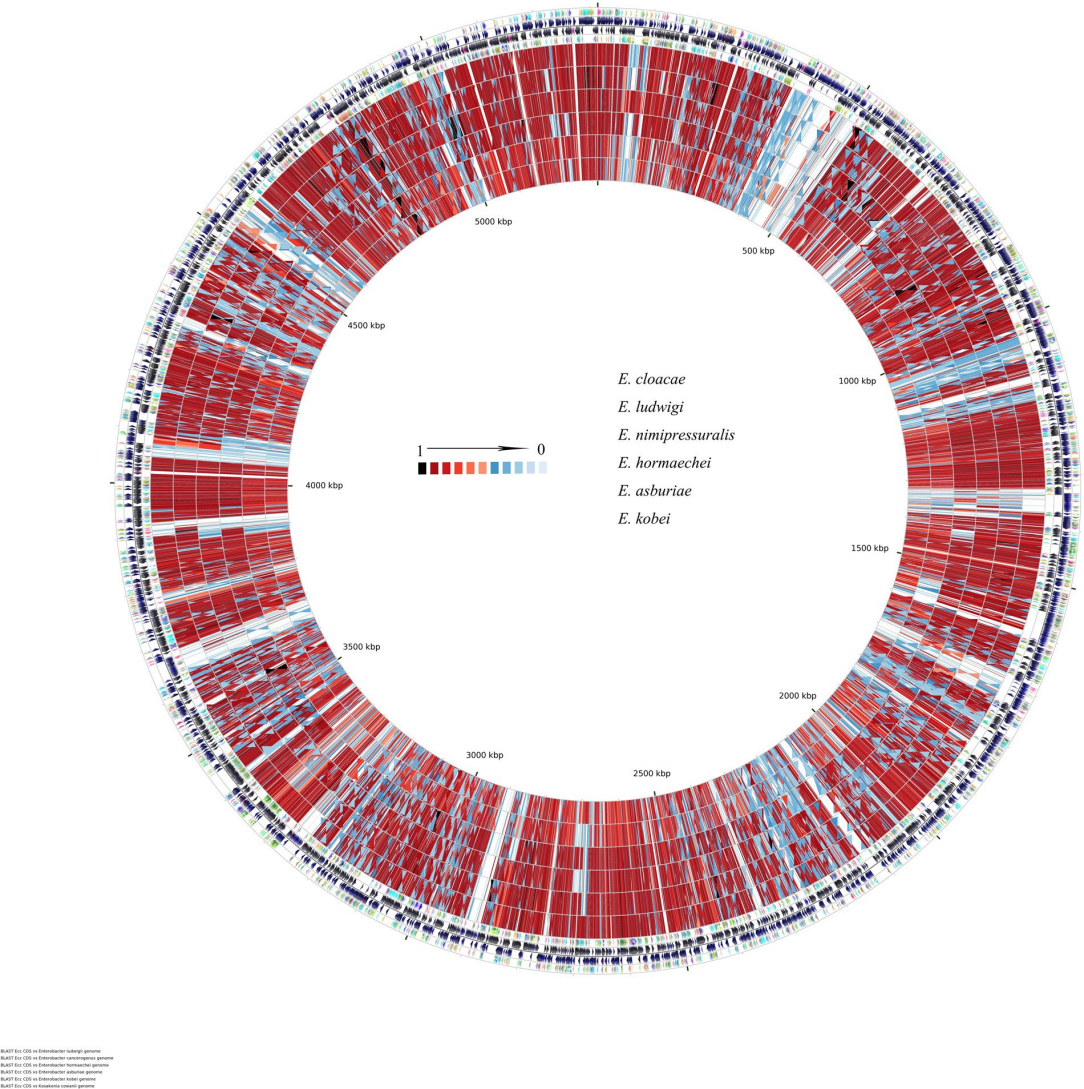
The circular visualization of the Ecc strains including *E. cloacae* subsp. *cloacae* and *E. cowanii* as reference strains. The map consists of six separate circles: 1 represents the reference, while the remaining 5 are the comparison genomes. The outermost ring depicts the following features: 1. COG Functional group based on COG for coding sequence of forward strand; 2. Sequence features of forward strand; 3. Sequence features of reverse strand; 4. Functional group based on COG for coding sequence of reverse strand. The remaining circles represent the similarity which was detected via BLAST(blastp).

For comparative genome-wide analysis, the sequence-based comparative analysis results were obtained for all species with the RAST server and CGview genome-wide analysis tools. The circular genomes of the Ecc strains, including *E. cloacae* subsp. *cloacae* ATCC 13047 and *E. cowanii* as reference strains, are depicted in Fig. 1. The circular genomes of the Ecc strains exhibited the coding sequence (CDS), ORF, GC content, number of RNAs and GC skew (Fig. 1), where the inner six rings represented the CDS and the number of RNAs (tRNA, rRNA and sRNA) on the forward and reverse strands. The visualization of the entire genome showed high similarity levels (>90%) and depicted numerous sites of latent deletion/insertion events in the genome of the Ecc strains (Fig. 1). It was further revealed that all Ecc strains, including *E. cloacae subsp. cloacae*, have 80–90% similarity in their core genome (Fig. 1). Various genes were classified as hypothetical, which is consistent with previous studies on genome-wide analysis of other species. In genomics, the comparisons and characterizations of functional elements depend on the identification of the natural selection footprints.

### Genome wide gene prediction and analysis of virulent genes

Comparative genome analysis helps to investigate natural evolutionary processes such as genetic drifts, virulence factors, drug resistance genes and mutations^9,10^. Moreover, antimicrobial resistance has evidently damaging effects in infectious diseases and is the main threat of superbugs around the globe^9,10^. By using reference sequences as well as virulence genes annotated with the VFDB filtered database, we analysed 5 Ecc genomes. A large number of functional genes contributing to drug resistance, stress resistance, general physiology and virulence of the bacteria were identified in all Ecc genomes studied (Tables S1, S2 and S3).

Several genes responsible for antibiotic resistance were also retrieved by computational analysis (Table S2) in Ecc, including β-lactamase-encoding, ABC transporter and efflux system genes (Table S2). In the past two decades, bacteria producing extended-spectrum β-lactamase (ESBL) have been identified mostly from humans and animals samples^11^. ESBLs confer resistance to extended-spe ctrum cephalosporins (ESCs, e.g., ceftriaxone, cefpodoxime, ceftiofur, ceftazidime) and monobactams and often express resistance to non-β-lactam antimicrobials, leaving limited therapeutic options. Moreover, recent studies have shown that in addition to direct killing effects, exposure to antibiotics at subinhibitory concentrations could cause an increase or reduction in the virulence of human and animal bacterial pathogens. The prediction of resistance genes in this pool revealed that Ecc strains are potentially threatening^11^.

The bacterial response is activated by potentially damaging conditions that result in alterations in gene activity and gene expression. Bacterial growth and survival are also dependent on changes in osmotic pressure^12^. We conducted a genome-wide survey and found that several key elements were associated with the adaptation of Ecc strains to various niches. Ecc bacteria contain multiple osmoregulatory systems (Table S3) to ensure optimal metabolic fitness^12^. proP is a proton symporter that senses and responds to osmotic shifts by bringing osmolytes such as glycine betaine, proline, pipecolic acid, stachydrine, taurine and ectoine into the cell. ProP is both an osmoregulator and an osmosensor that plays a role in the osmoregulatory response of bacteria^12^.

Pathogenicity-associated genes, such as T1SS, T2SS, T3SS, T4SS, and T6SS, which encode secretion system proteins and play essential roles in causing diseases not only in plants but also in animals, were identified^13,14^. Bacterial genomes have evolved a number of complicated nanomachines, such as T6SS, that aid in the transfer of a variety of virulence determinants across the bacterial cell membrane. In recent years, substantial development has been made to understand gram-negative bacterial secretion systems (I-VI) at both the molecular and structural levels^15,16^. We identified T1SS, T2SS, T4SS and T6SS in five Ecc strains. Notably, the T3SS, which is a key component of the secretion machinery, was not encoded by any strain in this study. The T3SS in the bacterial structure helps gram-negative pathogens invade the host with an exclusive mechanism of virulence and enables them to bypass the extracellular barriers^16–18^.

Gram-negative bacteria secrete type 1 secretion systems via a translocator comprising three proteins: (1) 2 cytoplasmic membrane proteins, i.e., an adaptor or a so-called membrane fusion protein, (2) an ATP-binding cassette (ABC) and (3) a specific outer membrane protein (OMP)^19^. We identified a set of type 1 secretion system-associated genes, including hlyB, tolC, macA and macB (Table S1), by BLAST analysis on the RAST server against each genome by utilizing virulence factor databases such as VFDB, UniProt and the literature. T1SSs were also reported to be activated by C-terminal secretion signals by initial interactions with ABC exporter proteins, resulting in secretion complex assembly by triggering specific protein interactions among ABC exporter, OMP, and MFP type 1 secretion system proteins. ABC exporter and T1SS proteins provide substrate specificity for system activation, leading to the secretion of HlyA, proteases and C-terminal secretion signals upon activation^16^.

The T2SS, which is encoded by 12 core genes, including T2SD, T2SE, and T2SG, is a major pseudophilin along with some minor pseudophilin genes, such as T2S H, J, I, and K, which play a significant role in ATPase attachment to the cell inner membrane. Additionally, T2S apparatus comparing F, M, and L, the inner (trans) membrane-associated proteins, and T2S O, the pre-pseudopilin peptidase/methyltransferase that also acts on type 4 prepilin, were identified. The T2S C protein of the T2SS is responsible for specific substrate identification and interaction for secretion^20^. Apart from these 12 core genes, other genes, such as T2S A, T2S S, T2S B and T2S N, were also found in some Ecc strains (Table S1). Based on the genome annotation carried out in this study, it was noted that the T2SS and its components were absent in *E. kobei, E. hormaechei*, and *E. ludwigii*, while *E. nimipressuralis* and *E. asburiae*, along with the reference strain, encode entire T2SS components with the exception of the *gspE* genes. The absence of T2SS genes indicates that these strains may not be pathogenic or may use other bacterial systems to invade the host^20^. In addition, these strains were isolated from clinical specimens, but there is a lack of experimental data to link the absence of T2SS with the pathogenicity of these strains.

Many gram-negative bacterial pathogens, plants and animal symbionts possess type III secretion systems comprising 20 different cytoplasmic, integral and outer membrane soluble proteins^21,22^. Ecc genomes lack any such genes, yet Ecc species possess all the genetic material needed for the cellular pathway controlling flagellum assembly. The Ecc strains reported in our study depend on flagellum-like proteins instead of all components of the T3SS.

The type IV secretion system (T4SS) is evolutionarily related to the conjugation system of bacteria^23^. T4SSs are categorized into type 4 A (T4ASS) or type 4B (T4BSS), depending on the structural components resembling either the *Agrobacterium tumefaciens VirB/D4* complex or the IncI plasmid conjugation system, respectively^23^. T4SS A and B both directly transfer effector proteins to the host cytosol through a central pore. In this study, two sets of T4ASSs were found in the Ecc genomes (Table S1), including all of the genes of the *virB/D4* complex but not *virB7*^24^. The low GC content and the presence of T4SS may be linked with horizontal gene transfer (HGT), which helps bacteria adapt to environmental changes and acquire antibiotic resistance. The Ecc strains studied here may be able to arbitrate HGT, which might facilitate the adaptation of Ecc strains to various environmental changes leading to antibiotic resistance.

**Figure 2:**
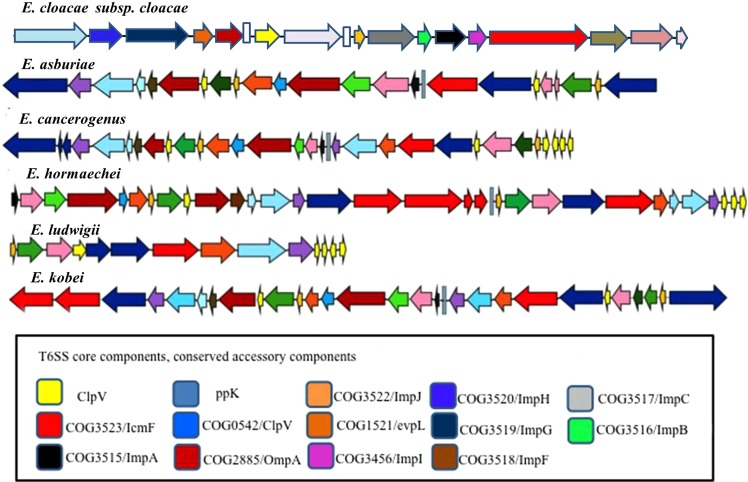
Genetic organization of various T6SS gene clusters in Ecc strains. The arrows show the genes while the direction of transcription is represented based on the direction of the gene. The gene components of conserved T6SSs are shown in red while the rest of the various colours depict non-conserved genes.

The type VI secretion system (T6SS) transfers effector proteins into host cells (either eukaryotes or prokaryotes) in a contact-dependent manner. The major targets of T6SS antibacterial effectors include the cell wall, membrane or nucleic acid^25^. The T6SS comprises at least 14 subunits forming the core machinery apparatus, and these subunits are encoded by genes in the imp operon. Overall, all *imps*, as well as *hcp* components, were present in the Ecc 5 type strains studied here. *icmf, impaK, impM* and *lipoprotein/VasD* were exceptionally absent in *E. kobei* and in *E. ludwigii* (Table S1). It is also interesting to note that *E. asburiae, E. nimipressuralis* and *E. hormaechei* encode two loci of T6SS clusters. The genetic organization of each T6SS of the Ecc strains is shown in Fig. 2.

The functions of T6SS genes are diverse, including virulence, microbe-microbe interactions, and host-pathogen interactions. Moreover, the ATPase activity of the intracellular multiplication protein F family protein (*icmF*) energizes the T6SS system. It seems that the absence of this protein may have effects on the entire T6SS system in *E. kobei* and in *E. ludwigii*, or these species may adopt other pathways. It will be interesting to explore the function of *icmF* in these species by generating *icmF* gene mutants. Moreover, *E. asburiae, E. cancerogenus* and *E. hormaechei* were exceptions because these strains contained two T6SS loci in the genomes and lacked some core and conserved components. This feature of T6SSs in Ecc has been reported previously in the genomes of Yersinia pseudotuberculosis and Burkholderia pseudomallei, which harbour six and four T6SS loci, respectively^25–27^. Similar to B. pseudomallei and Y. pseudotuberculosis, *E. asburiae, E. cancerogenus*, and *E. hormaechei* encode two loci that may be differentially regulated and possess different functions.

The regulation of the T6SS is controlled at multiple levels. The T6SS gene cluster subset encodes orthologues of the forkhead-associated (*FHA*), cognate phosphatase *pppA*, and serine/threonine kinase *ppkA* domain–containing proteins. This indicates an association of a threonine phosphorylation (*TPP*) regulatory pathway in bacteria^25^. These three major components of regulatory pathways are encoded within the *imp* operon. In all five Ecc genomes, at least one regulatory pathway was identified, including protein serine/threonine phosphatase *prpC* (Table S1).

Apart from the 14 core genes, many additional genes, such as 9 genes of the *hcp* operon, e.g., *clpB* and *vgrG*, are also required for system assembly and function^26^. Valine-glycine repeat protein G (*vgrG*) and hemolysin-coregulated protein (hcp) are known as T6SS hallmarks. Hcp is also considered to be an important chaperone for T6SS effectors, preventing their degradation, and *hcp* is secreted together with these effectors. In addition to its structural function in the T6SS, *hcp* is thought to be a secretory protein with multiple functions involved in bacterial invasion, cell adherence, colonization, and competition, as well as intracellular reproduction, in different bacteria^27^. In our study, *vgrG/hcp* genes were found as an orphan component. The presence of *hcp* and v*grG* proteins along with the previously identified T6SS locus suggests that Ecc encodes a functional T6SS.

Notably, *E. nimipressuralis* CIP 104980 (clinical), *E. hormaechei* ATCC 49162 (sputum of a male patient), *E. ludwigii* EN-119 (clinical) and *E. kobei* JCM 8580 (clinical) sequenced in this study encode well-defined T6SSs containing *vgrG* and *hcp* proteins as orphan components. Several studies have reported the niche, antibiotic resistance and virulence of *E. kobei* and *E. ludwigii*, which are most commonly isolated from human clinical specimens^28–30^. Although these strains are most commonly associated with clinical specimens, variations in loci containing regulatory proteins such as *prpC* or *prpK*, *hcp* and *vgrG* have been observed, showing that these strains encode functional T6SSs.

### Evolutionary analysis of Ecc strains

**Figure 3:**
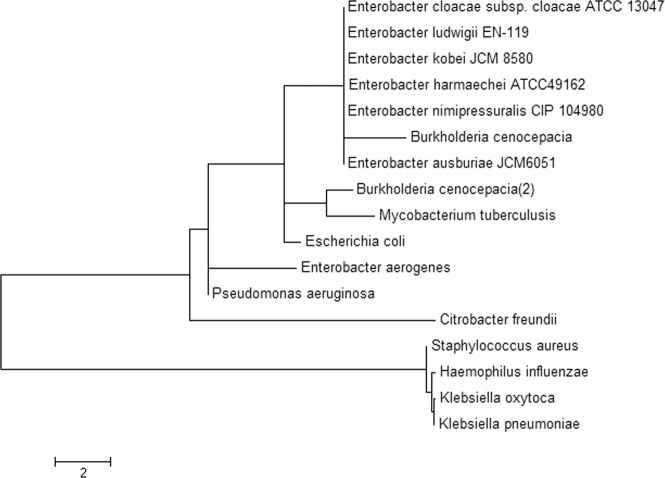
rpoB-based phylogenetic analysis of *Enterobacter cloacae complex* strains and other selected species of opportunistic bacteria.

The full-length *rpoB* sequences of five Ecc strains, namely, *E. kobei, E. hormaechei, E. ludwigii*, *E. nimipressuralis* and *E. asburiae*, and eight other bacterial species from various sources, particularly well-known pathogenic strains, were evolutionarily characterized. The sequence comparison revealed that five Ecc strains, i.e., *E. kobei, E. hormaechei, E. ludwigii E. nimipressuralis* and *B. cenocepacia*, formed a single clade, and *B. cenocepacia*, an opportunistic human pathogen, was also in the same clade. Phylogenetic affiliation of the Ecc strains with *B. cenocepacia* and other species, such as *E. coli, Pseudomonas aeruginosa*, and *Mycobacterium tuberculosis*, showed that the Ecc strains might encode similar pathogenic features (Fig. 3).

### Quantitative expression and analysis of T6SS genes

In this study, the structural genes (impA-impE, impG-impK, impM and prpC) of the 5 Ecc strains mentioned in Table 2 showed notable expression levels of activation in ASM culture conditions that mimicked *in vivo* conditions compared to LB medium, which was used as a control. This notable expression was observed in all Ecc-type strains with little variation. The imp operon is a conserved part of almost all T6SSs and actively participates in mediating *hcp* secretion^9,30^. The high expression of the imp operon and *prpC* under *in vivo* mimicking conditions revealed that the imp operon seems to play a very active role in virulence and host-microbe interactions and is fully functional. It has been determined that *icmF* and *clpB* are powerful T6SS hallmark proteins and that *hcp* exhibits ATPase activity^10^. Significantly, the high expression of *icmF* in all Ecc type strains showed that the secretion of the T6SS is powered by icmF. Furthermore, the absence of *icmF* was also verified by our real-time qPCR analysis, and its absence highlights that *clpB* may play a key role in the secretion of the T6SS imp operon in the absence of *icmF* in *E. kobei* and *E. ludwigii*.

**Table 2:** Comparative gene expression profile of type six secretion systems in *Enterobacter cloacae complex* in ASM and LB medium optimized at log phase.

	E. nimipressuralis		E. ludwigii		E. hormaechei		*E*. asburiae		*E. kobei*	
Change in Expression Relative to the Expression in vitro(LB media) under the Following Conditions										
Name of gene	*LB*	*ASM*	*LB*	*ASM*	*LB*	*ASM*	*LB*	*ASM*	*LB*	*ASM*
ClpB	40.1	1.5	7.9	10.2	1.6	2.2	3008.4	40.1	1.5	107.9
icmf	1.4	321.3	0.02	0.0	10.4	211.5	28.6	219.0	0.0	0.01
impA	23.1	93.5	8.6	101.5	6.4	176.2	1.7	213.2	3.4	109.5
impB	12.8	101.2	5.0	89.8	0.12	211.4	3.2	143.8	9.21	131.0
impC	7.2	112.7	7.2	79.5	3.7	289.1	7.6	501.3	7.4	108.3
impD	0	0	4.6	91.9	7.9	5.1	7.4	401.7	4.1	95.9
impE	17.2	79.0	6.5	103.9	6.0	191.3	4.2	501.2	9.3	98.6
impG	16.4	86.8	5.3	237.6	5.8	8.3	9.2	121.4	5.4	187.3
impH	12.3	77.5	6.2	172.2	5.8	261.4	3.2	202.4	3.6	216.1
impI	21.1	112.6	12.1	94.9	9.7	302.1	5.1	201.9	3.1	198.3
impJ	23.2	93.5	9.8	101.8	6.9	212.4	7.4	401.1	0.5	267.7
impK	9.0	89.8	0.0	0.0	5.3	98.8	5.6	102.0	0.0	0.0
impM	13.7	73.4	0.8	0.01	9.6	114.2	3.8	203.2	0.0	0.0
PrpC	13.8	91.3	19.3	102.9	2.1	209.3	4.1	123.8	0.2	191.0
VasD/Lip	9.8	2.7	0.0	0.1	4.2	231.8	17.7	5.1	0.0	0.21

The changes in gene expression or variations were two-fold higher than those in the control (*in vitro* or LB medium conditions).

## Conclusions

Ecc genome-wide sequence analysis reveals key insights into the potential spread and evolution of antimicrobial resistance and virulence, including the T6SS, efflux system and environmental stress-related genes. The literature shows that these genes might play a significant role in either increasing the general fitness (iron uptake system and protease) or the competitiveness of Ecc bacterial species, which help them to better survive in diverse environments. The high gene expression of the T6SS in ASM medium revealed that the T6SS might be a crucial determinant and indispensable for the survival of Ecc strains in diverse environments. Moreover, the differences in gene expression profiles also indicate that ASM could be considered an alternative model system for Ecc instead of rats or humans. The Ecc genome sequence data have provided us with a real-time patient management resource for genome-scale antimicrobial resistance gene prediction. Furthermore, RNA sequencing and genetic engineering will be imperative for the in-depth investigation of the pathology and niche adaption of these strains.

## Materials and Methods

### Culturing and genomic DNA extraction

The information about bacterial species/strains sequenced in this study is given in Table 1; the genome of *E. asburiae* was reported previously, while genome-wide analysis of the rest of the strains focusing on sigma factors was reported in our previous study^8,31^. Strains were cultured in LB medium and allowed to grow until mid-logarithmic phase at 30 °C in a shaking incubator. DNA was extracted using a genomic DNA isolation kit (Promega, Madison, WI). The concentration and quality of the DNA were determined by a Nanodrop 2000 (Thermo Fisher Scientific Germany).

### Genome sequencing and assembly

PacBio sequencing technology (Pacific Biosciences, USA) was used to sequence the whole genome of four Ecc strains. The PacBio RS II sequencer was equipped with Sequence Runs for four single-molecule Real-Time (SMRT) cells and a movie time of 120 minutes/SMRT cell. Adapter trimming and read filtering were conducted with SMART Analysis (V2.2) using default parameters. The 600 Mb postfiltered data (approximately 80X coverage with an average read length of 7 kb) were taken for assembly. The Hierarchical Genome Assembly Process (HGAP) assembly protocol was used to perform the *de novo* genome assembly equipped with the SMRT Analysis packages (V 2.2).

### Genome annotation and analysis

All four genomes were annotated using the RAST annotation system using SEED viewer for the prediction of rRNAs, tRNA, coding genes and GC content. Kyoto Encyclopedia of Genes and Genomes (KEGG) and Clusters of Orthologous Groups of proteins (COGs) were used for the classification of the predicted genes, i.e., virulent genes, various secretion systems, drug resistance genes and different pathways. The SignalP server and TMHMM server were used to predict the signal peptides and transmembrane helices in genes^32–34^.

### Comparative genomic analysis

The genome alignment of Ecc strains was conducted using the MAUVE software package^34^, which is employed to generate alignments of multiple genomes to look at the highly similar subsequences, evolutionary events such as inversions, and rearrangements and to reveal the correct global alignment. The genomes were further compared using the CGView tool^35,36^. A circular genomic map of the Ecc strains was generated using CGView, which presents circular genomes in a graphical map, resulting in base composition plots, sequence features and analyses of the GC skew, GC content and number of RNAs.

Comparison of the genomes of *E. nimipressuralis* CIP 104980, *E. hormaechei* ATCC 49162, *E. asburiae* JCM 6051, *E. ludwigii* EN-119 and *E. kobei* JCM 8580 was carried out by using Circos 36. Muscle was used to generate the sequence alignment. Conserved regions were trimmed by using trimAl^37^.

### Genome-wide prediction and analysis of virulent genes

The existence of putative virulence-associated genes in the draft genomes of the Ecc strains isolated from various niches was analysed using the reference strain *E. cloacae subsp. cloacae* ATCC 13047 with RAST and SEED viewer. The presence or absence of virulent and drug-resistant coding sequences such as T1 to T6SS and efflux drug-resistant genes in the five Ecc genomes were retrieved using *E. cloacae subsp. cloacae* ATCC 13047 genes as bait sequences following independent confirmation by performing nucleotide BLAST analysis at NCBI as well as BioEdit. The genes with query coverage higher than 70% and similarities higher than 50% were taken as homologs. Using the RAST annotation output, the presence of putative known pathogenicity and drug resistance-associated genes were further investigated.

Furthermore, we conducted an extensive computational analysis including BLAST and BLASTP for the prediction of drug resistance genes in the five Ecc genomes. Moreover, the antibiotic resistance gene database (ARDB)^38,39^ was also used to verify the drug resistance gene dataset to predict virulence and drug resistance genes in the five type strain genome sequences in our study.

To explore the bacterial secretion system, we followed the methods of Li *et al*., T346Hunter and SecReT6^40,41^, where the various secretion systems and the nominal adequate number of components of the secretion system have been explained in detail. Briefly, among other things, the method for identifying the secretory genes following relevant information for secretion system analysis was specified^40,41^. For example, (1) the secretory system is encoded in a single locus or in a multiple locus, (2) the core components (essential and ubiquitous) must be encoded, and (3) the components are defined as accessory if they are accessory or poorly conserved sequences, and the function of these sequences may be essential. Considering this information and using reference genes of *E. cloacae subsp. cloacae* ATCC 13047, BLASTP, BLASTN, TBLASX, HHpred, Phyre2, and SecReT6 as well as T346Hunter-based systematic analysis of the genetic architecture and gene contents of the secretion system was conducted^42^.

### Evolutionary analysis of ecc based on the rpoB gene

The genetic diversity and evolutionary analysis of Ecc, i.e., *E. nimipressuralis* CIP 104980, *E. hormaechei* ATCC 49162, *E. asburiae* JCM 6051, *E. ludwigii* EN-119 and *E. kobei* JCM 8580, were analysed by reconstructing the phylogenetic tree based on the multiple sequence alignment data of rpoB, which encodes the β subunit of the bacterial RNA polymerase gene, which is used to analyse microbial diversity. MUSCLE, a comprehensive and powerful tool for next-generation sequence and molecular biology analysis, was used for nucleotide sequence alignment of rpoB genes. Evolutionary relationships among five Ecc strains were constructed using the MEGA 7 and CLUSTAL OMEGA tools in the form of an evolutionary tree. MEGA 7 was set with default parameters to build a tree with additional settings, including UPGMA, the neighbour-joining method, and minimum evolution method, with default preferences, including the statistical method, Poisson model and phylogeny reconstruction.

### Preparation of ASM and quantitative real-time PCR

T6SS quantitative gene expression analysis was conducted in control medium (LB) and artificial sputum medium (ASM). ASM is a culture medium containing the components of cystic fibrosis (CF) patient sputum, including amino acids, mucin and free DNA. Dozens of studies revealed that ASM could be used to mimic the host, particularly respiratory conditions, and as an alternative model to achieve reproducible culture conditions to study drug resistance, virulence and niche adaptation^43^. ASM was used to assess not only its efficacy as a medium but also to examine the gene expression profile of the T6SS by real-time PCR analysis. The lists of primers designed by using Primer 3 are listed in Table S4. ASM was prepared as described by Lawal *et al*.^43^. The inoculated Ecc culture was harvested at stationary phase. RNA was extracted using the RNAeasy Mini Kit (Qiagen, Germany); the cDNA library was prepared using a kit (Thermo Fischer Scientific, USA), and quantitative PCR analyses were conducted using PikoReal Real time PCR (Thermo Fischer Scientific, USA). The fold change in gene expression was determined by the average threshold cycle (Ct). The 2^−ΔΔCt^ method was used for relative quantification of the T6SS genes in the Ecc strains.

## Supplementary information

Table S1

Table S2

Table S3

Table S4.

## Data Availability

The genome sequence data for each strain are available at the following accession numbers in the NCBI and GenBank databases: *E. Ludwigii* EN-119 (CP017279), *E. hormaechei* ATCC 49162 (MKEQ. 00000000), *E. nimipressuralis* CIP104980 (MKER00000000), *E. kobei* JCM 85580 (MKXD00000000)^T^ and. *E. asburiae* JCM 6051 (CP011863–CP011867). The annotation data and analysis are available from the corresponding author.

## References

[1] Hormaeche E, Edwards PR (1958). Observations on the genus Aerobacter with a description of two species. Int. Bull. Bacteriol. Nomen. Taxon..

[2] Paauw A (2008). Genomic diversity within the *Enterobacter cloacae complex*. Plos one.

[3] Streit JM, Jones RN, Sader HS, Fritsche TR (2004). Assessment of pathogen occurrences and resistance profiles among infected patients in the intensive care unit: report from the sentry Antimicrobial Surveillance Program (North America, 2001). Int. J. Ant. Agen..

[4] Harbarth S, Sudre P, Dharan S, Cadenas M, Pittet D (1999). Outbreak of *Enterobacter cloacae* related to understaffing, overcrowding, and poor hygiene practices. Inf. Con. Hos. Epid..

[5] Ren Y (2010). Complete genome sequence of *Enterobacter cloacae* subsp. *cloacae* Type strain ATCC 13047. J. Bact..

[6] Garcia-Gonzalez T (2018). *Enterobacter cloacae*, an emerging plant-pathogenic bacterium affecting chili pepper seedlings. Plant Pathol. J..

[7] Harada K (2017). Phenotypic and molecular characterization of antimicrobial resistance in Enterobacter spp. isolates from companion animals in Japan. Plos One..

[8] Zhu B, Li O, Hussain A, Ibrahim M (2017). High quality genome sequence of human pathogen *Enterobacter asburiae* type strain 1497 78T. J. Glob. Antimicrob. Resist..

[9] Mezzatesta ML, Gona F, Stefani S (2012). *Enterobacter cloacae* complex: clinical impact and emerging antibiotic resistance. Future Microbiol..

[10] Hoffmann H, Roggenkamp A (2003). Population Genetics of the Nomenspecies *Enterobacter cloacae*. Appl. Env. Microbiol..

[11] Singh T (2019). Transcriptome analysis of beta-lactamase genes in diarrheagenic *Escherichia coli*. Sci. Rep..

[12] Wood JM (2015). Bacterial responses to osmotic challenges. J. Gen. Physiol..

[13] Green ER, Mecsas J (2016). Bacterial secretion systems: an overview. Microbiol. Spect..

[14] Kirzinger MW, Nadarasah G, Stavrinides J (2011). Insights into cross-kingdom plant pathogenic bacteria. Genes..

[15] Costa TR, Felisberto RC, Meir A, Prevost MS, Redzej A (2015). Secretion systems in Gram-negative bacteria: structural and mechanistic insights. Nat. Rev. Microbiol..

[16] Delepelaire P (2004). Type I secretion in Gram-negative bacteria. Biomembranes..

[17] Nivaskumar M, Francetic O (2014). Type II secretion system: a magic beanstalk or a protein escalator. Mol. Cell Res..

[18] Rondelet A, Condemine G (2013). Type II secretion: the substrates that won’t go away. Res. Microbiol..

[19] Holland IB, Schmitt L, Young J (2005). Type 1 protein secretion in bacteria, the ABC-transporter dependent pathway. Mol. Membr. Biol..

[20] Minamino T, Namba K (2008). Distinct roles of the FliI ATPase and proton motive force in bacterial flagellar protein export. Nature..

[21] Magdalena J (2002). Spa32 regulates a switch in substrate specificity of the type III secretion of Shigella flexneri from needle components to Ipa proteins. J. Bacteriol..

[22] Abby SS, Rocha EP (2012). The non-flagellar type III secretion system evolved from the bacterial flagellum and diversified into host-cell adapted systems. PLoS Gen..

[23] Voth DE, Broederdorf LJ, Graham JG (2012). Bacterial type IV secretion systems: versatile virulence machines. Future Microbiol..

[24] Wallden K, Rivera-Calzada A, Waksman G (2010). Type IV secretion systems: versatility and diversity in function. Cell Microbiol..

[25] Records AR (2011). The type VI secretion system: a multipurpose delivery system with a phage-like machinery. Mol. Plant Microbe. Interact..

[26] Esser D (2016). Protein phosphorylation and its role in archaeal signal transduction. FEMS Microbiol. Rev..

[27] Blair JM, Webber MA, Baylay AJ, Ogbolu DO, Piddock LJ (2015). Molecular mechanisms of antibiotic resistance. Nat. Rev. Microbiol..

[28] Peng P (2016). Roles of Hcp family proteins in the pathogenesis of the porcine extra intestinal pathogenic *Escherichia coli* type VI secretion system. Sci. Rep..

[29] Suzuki S, Horinouchi T, Furusawa C (2014). Prediction of antibiotic resistance by gene expression profiles. Nat. Commun..

[30] Jousset, A.B. *et al*. False-positive carbapenem-hydrolyzing confirmatory tests due to ACT-28, a chromosomally-encoded AmpC with weak carbapenemase activity from *Enterobacter kobei*. *Antimicrob. Agents Chemother*. **25**, 63(5) (2019). 10.1128/AAC.02388-18 PMC6496075 30783006

[31] Nazir F (2018). Genetic Diversity and Functional Analysis of Sigma Factors in Enterobacter cloacae Complex Resourced From Various Niche. Evol. Bioinform. Online.

[32] Overbeek R, Olson R, Pusch GD, Olsen GJ, Davis JJ (2014). The SEED and the Rapid Annotation of microbial genomes using Subsystems Technology (RAST). Nucl. Acids Res. (Database Issue).

[33] Petersen TN, Brunak S, Von Heijne G, Nielsen H (2011). SignalP 4.0: discriminating signal peptides from transmembrane regions. Nat. Methods..

[34] Darling ACE, Mau B, Blattner FR, Perna NT (2004). Mauve: multiple alignment of conserved genomic sequence with rearrangements. Genome Res..

[35] Stothard P, Wishart DS (2005). Circular genome visualization and exploration using CGView. BMC Bioinform..

[36] Naquin D (2014). CIRCUS: a package for Circos display of structural genome variations from paired-end and mate-pair sequencing data. BMC Bioinform..

[37] Capella-Gutierrez S, Silla-Martinez JM, Gabaldon T (2009). trimAl: a tool for automated alignment trimming in large-scale phylogenetic analyses. Bioinformatics.

[38] Liu B, Pop M (2009). *ARDB-Antibiotic Resistance Genes Database*. Nucl. Acid Res..

[39] Chen L (2005). VFDB: a reference database for bacterial virulence factors. Nucl. Acid Res..

[40] Martinez-Garcia PM, Ramos C, Rodriguez-Palenzuela P (2015). T346Hunter: A Novel Web-Based Tool for the Prediction of type III, type IV and type VI Secretion Systems in Bacterial Genomes. Plos one..

[41] Li J (2015). SecReT6: a web-based resource for type VI secretion systems found in bacteria. Plos one..

[42] Kumar S, Stecher G, Tamura K (2016). MEGA7: Molecular Evolutionary Genetics Analysis version 7.0 for bigger datasets. Mol. Biol. Evol..

[43] Lawal O (2018). BreathDx consortium. TD/GC-MS analysis of volatile markers emitted from mono- and co-cultures of *Enterobacter cloacae* and *Pseudomonas aeruginosa* in artificial sputum. Metabolomics..

