# Supplementary material for: Genome-wide Analysis of Four Enterobacter cloacae complex type strains: Insights into Virulence and Niche Adaptation
Source: Sci Rep. 2020 May 18;10:8150. doi: 10.1038/s41598-020-65001-4 (PMC7235008; doi:10.1038/s41598-020-65001-4)
Supplement: Supplementary file 4 — Table S4. [file 41598_2020_65001_MOESM4_ESM.docx]

**Genome wide Analysis of Four *Enterobacter cloacae* complex type strains: Insights into Virulence and Niche Adaptation**

**Areeqa Mustafa^1,2^, Muhammad Ibrahim^1,2^, Muhammad Asif Rasheed^2^, ^2^Sumaira Kanwal, Annam Hussain^2^, Asma Sami^2^, Raza Ahmed^3^, Zhu Bo^1^**

^1^School of Agriculture and Biology, Shanghai Jiao Tong University/Key Laboratory of Urban Agriculture by Ministry of Agriculture of China, Shanghai 200240, China

^2^Genomics and Computational Biology Laboratory, Department of Biosciences, COMSATS University Islamabad, Sahiwal Campus, Sahiwal, Pakistan.

^3^Department of Biotechnology, COMSATS University Islamabad, Abbottabad Campus, Pakistan.

**Suplementry Tables**

**Table S1**. The *In silico* identified secretion system of *Eenterobacter cloacae* complex: Sheet 1. Type 1 secretion system: Sheet 2. Type two section system: Sheet 3. Type 4 section system: Sheet 4. Type 6 secretion system.

**Table S2.** Computational prediction and characterization of antibiotic resistance and multidrug resistnce components in genus *Enterobactera*

**Table S3.** Computational prediction and characterization of Environmnetal stress related genes in e genus *Enterobactera*

**Table S4.** List of Primer used in this study

| **Name of Genes** | **Primers 5’ to 3’** |
| --- | --- |
| ClpB | CGCCAGATGGTGGATATTCT  TGTTCAAACTCCCCCTTCAC |
| icmf | CTTTGCCCGCTCTAACTTTG  ATACCGGCTTCCTGGAAAAT |
| impA | GTAGAAAAGCTCGCCACCAG  CCTCCAGCAGTGGATAGAGC |
| impB | CGACAATTTCGACGAAGTCA  TATCTTCCAGCATCCGTTCC |
| impC | ACTGAAACCGACGAGATGCT  TCACCACCAAACTGACCGTA |
| impD | TCACACCGGTTGGACTGATA  GATTCGCGCATATTCAACCT |
| impE | GCTTCGACTGGTTGATGGAT  AGAAGCGGTCTTCAACCTGA |
| impG | AACGCTGCCTTGTTATCCAC  TCCAGCGTCACTTCAATCTG |
| impH | AACGCTGCCTTGTTATCCAC  TCCAGCGTCACTTCAATCTG |
| IMPI | CCTCAAGGGTGAGTTCCGTA  CGCAAGTCATCGAAACTGTC |
| impJ | GGGGCTTTCTTGATGTTGAA  ACCACCTTTTCGTTGGTGAG |
| impK | CTCGGCAGTTACCGATCATT  CGACCACTATCACGCTCAGA |
| impM | GTCGCTATTTCCCGCTGAC  ATCAGCGATTGTTCGATGC |
| PrpC | GGGAGAAATGGAATGAAGCA  CGATATGGGCAACAGAAACC |
| VasD/Lip | GTCATGACATTGACGCGAAC  TGATCACCCCTTCTTTTTCG |
